# Unveiling the Importance of Early Detection of Oral Mucosal Melanoma with Non-Invasive Imaging Techniques

**DOI:** 10.3390/diagnostics16071030

**Published:** 2026-03-30

**Authors:** Beatrice Bălăceanu-Gurău, Matteo Liberi, Francesco D’Oria, Giulio Foggi, Francesco Piscazzi, Chiara Tronconi, Mario Valenti, Gisele Gargantini Rezze, Milind Rajadhyaksha, Marco Ardigò

**Affiliations:** 1 Department of Oncologic Dermatology, “Elias” Emergency University Hospital, “Carol Davila” University of Medicine and Pharmacy, 050474 Bucharest, Romania; 2 Dermatology Unit, IRCCS Humanitas Research Hospital, 20089 Rozzano, MI, Italy; matteo.liberi@st.hunimed.eu (M.L.); francesco.doria@humanitas.it (F.D.); giulio.foggi@humanitas.it (G.F.); francesco.piscazzi@humanitas.it (F.P.); mario.valenti@hunimed.eu (M.V.); marco.ardigo@hunimed.eu (M.A.); 3 Department of Biomedical Sciences, Humanitas University, 20072 Pieve Emanuele, MI, Italy; 4 Oncology Unit, IRCCS Humanitas Research Hospital, 20089 Rozzano, MI, Italy; maria_chiara.tronconi@humanitas.it; 5 Dermatology Department, University of Trieste, 34127 Trieste, TS, Italy; ggrezze@hotmail.com; 6 Dermatology Service, Memorial Sloan Kettering Cancer Center, New York, NY 10065, USA; rajadhym@mskcc.org

**Keywords:** oral mucosal melanoma, non-invasive imaging techniques, dermoscopy, reflectance confocal microscopy, early diagnosis

## Abstract

Oral mucosal melanoma (OMM) is a rare and aggressive malignancy that differs markedly from cutaneous melanoma in terms of epidemiology, genetic characteristics, clinical presentation, and treatment response. Despite advances in understanding OMM pathogenesis and the development of novel therapeutic strategies, early diagnosis remains challenging due to its low prevalence, anatomically concealed locations, and frequent multifocality. This review emphasizes the importance of the early detection of OMM using non-invasive imaging methods—specifically dermoscopy and reflectance confocal microscopy (RCM)—and explores their potential role in guiding treatment decisions, preventing disease progression, and improving prognosis. A narrative review of the PubMed database was conducted using the terms “oral melanoma,” “oral melanoma dermoscopy,” and “oral melanoma reflectance confocal microscopy.” Seventy-two relevant review articles were included. In addition, two illustrative clinical cases from our practice are presented to demonstrate the diagnostic value of non-invasive imaging techniques. Although biopsy and histopathology remain the diagnostic gold standards, they are invasive, time-consuming, and may be poorly tolerated, particularly in patients with multifocal lesions. Dermoscopy and RCM provide real-time, high-resolution imaging that enables the detection of early tissue abnormalities not visible to the naked eye. These techniques show good correlation with clinical and histopathological findings, thereby enhancing diagnostic accuracy and facilitating follow-up without the need for repeated biopsies. In our cases, they were instrumental in identifying recurrence and guiding clinical management. However, several limitations should be considered, including restricted accessibility, anatomical constraints, and the requirement for specialized training and expertise. Non-invasive imaging techniques may support clinicians in the early recognition and evaluation of suspicious oral lesions; however, histopathologic examination remains essential for definitive diagnosis. Wider implementation and further technological refinement are needed to optimize their integration into clinical practice.

## 1. Introduction

Oral mucosal melanoma (OMM) is a rare and aggressive malignancy, accounting for approximately 0.8% of all melanomas and 8% of head and neck melanomas [1,2,3,4,5,6]. According to global cancer registries, it also represents about 0.26% of oral cavity cancers [1,2,3,4,5,6]. The annual incidence of OMM is approximately four cases per 10 million individuals, with a slight male predominance [3,7,8,9,10,11]. The highest incidence has been reported among Asian populations, particularly in Japan, where oral melanoma accounts for up to 7.5% of all melanomas, compared to less than 1% in Caucasian populations [3,7,8,9,10,11]. OMM predominantly affects adults, with very few cases occurring in individuals under 20 years of age [7,12]. The average age at diagnosis is approximately 56 years, with a reported range between 22 and 83 years [7,13,14,15,16].

The etiology of OMM remains largely unknown, and unlike cutaneous melanoma (CM), ultraviolet radiation is not considered a contributing factor [3,17,18,19,20]. Mechanical stress, including injury from ill-fitting prostheses, repetitive trauma, infection, and tobacco use, may influence the distribution of melanoma within the oral cavity and contribute to alterations in the mitogen-activated protein kinase (MAPK) signaling pathway observed in OMM [7,8,17,18,21]. In vitro studies have demonstrated that mechanical stimulation of melanocytes enhances MAPK signaling, which is associated with increased cell proliferation, invasiveness, and metastatic potential [7,8,18].

OMM originates from melanocytes located within the basal layer of the oral mucosal epithelium [2,7,22]. It is thought to arise from pre-existing pigmented lesions, such as melanocytic nevi or melanosis, from Hutchinson’s premalignant lentigo, or de novo from clinically normal mucosa [2,4,7,8,9,23]. OMM may present as pigmented or amelanotic lesions and is clinically classified into nodular, macular, or mixed types [2,17]. The most commonly affected sites are the hard palate and maxillary gingiva; however, other oral locations, including the tongue, buccal mucosa, mandible, and both upper and lower lips, may also be involved [2,7,9,10,24,25].

OMM is characterized by aggressive clinical behavior and a poorer prognosis compared with CM, primarily due to delayed diagnosis and the often asymptomatic nature of early lesions [2,3,12,18,21,26,27,28]. The reported 5-year overall survival rate is approximately 25% [2,5,8,12,26,29]. Independent prognostic factors include undifferentiated tumor cell morphology, vascular and perineural invasion, tumor necrosis, tumor thickness, and cervical lymph node metastasis [8,14]. Molecular markers such as the loss of p16 expression, nuclear BAP1 expression, and aberrant p53 expression have been associated with poorer outcomes, whereas Bcl-2 expression has been linked to a more favorable prognosis [8,30]. Notably, a study by Cui et al., including 706 patients, found no significant differences in survival among mucosal melanomas arising from different anatomical sites when matched for stage, prognostic factors, and molecular characteristics [31].

Despite advances in understanding OMM pathogenesis and the development of novel therapeutic strategies, early diagnosis remains essential for optimal patient management, as it significantly reduces the risk of metastasis and improves survival outcomes. Raising awareness among both dermatologists and dentists regarding the importance of routine oral cavity examination is therefore crucial [2,17,18,27,32]. Particular attention should be given to the inspection of the hard palate and maxillary gingiva, which represent the most common sites of involvement [27].

In clinical practice, the diagnosis of OMM remains particularly challenging because early lesions are often subtle, asymptomatic, and located in anatomical areas that are not routinely examined [14,33]. As a result, many cases are diagnosed at advanced stages, when regional or distant metastases may already be present [5,34].

Furthermore, the clinical presentation of oral pigmented lesions is highly heterogeneous and may overlap with a wide range of benign conditions, including oral melanosis, melanocytic nevi, amalgam tattoos, and drug-induced pigmentation [35,36,37]. These diagnostic challenges highlight the need for additional tools to assist clinicians in the early identification of suspicious lesions.

This review emphasizes the role of early diagnosis using non-invasive imaging techniques and their potential impact on treatment decision-making, with the aim of preventing disease progression and improving patient outcomes. To illustrate their clinical applicability, we present two cases from our practice in which dermoscopy and reflectance confocal microscopy (RCM) played a key role in diagnosis, follow-up, and management, thereby supporting informed clinical decision-making.

Nevertheless, these techniques have certain limitations, most notably restricted accessibility and the lack of standardization, which highlight the need for further research and technological development to optimize their integration into clinical practice.

## 2. Materials and Methods

This narrative review was conducted to summarize the current evidence regarding the use of non-invasive imaging techniques for the diagnosis and monitoring of oral mucosal melanoma. A literature search was performed in the PubMed database in January 2025 using the following keywords and combinations: “oral melanoma”, “oral mucosal melanoma”, “oral melanoma dermoscopy”, and “oral melanoma reflectance confocal microscopy”.

Articles published in English between January 2000 and December 2024 were considered. Titles and abstracts were screened to identify studies relevant to the objectives of the review, namely the role of non-invasive diagnostic techniques in oral mucosal melanoma, with particular emphasis on dermoscopy and reflectance confocal microscopy.

Articles addressing unrelated malignancies, non-oral melanomas, or topics not related to diagnostic imaging were excluded. Both review articles and selected original studies of particular relevance were considered to ensure a comprehensive overview of the topic. Case reports were excluded from the literature analysis; however, two illustrative cases from our clinical practice were included separately to demonstrate the clinical applicability of non-invasive imaging techniques.

A total of 72 review articles were included in the qualitative synthesis, while additional references were used to support background information and discussion.

## 3. Discussions

The clinical evaluation of pigmented lesions of the oral mucosa requires careful differentiation between malignant and benign conditions. A wide range of entities may mimic oral mucosal melanoma, including physiologic pigmentation, oral melanotic macules, melanocytic nevi, smoker’s melanosis, drug-induced pigmentation, and amalgam tattoos [5,35,36,37]. In addition, vascular lesions, Kaposi sarcoma, and certain pigmented variants of squamous cell carcinoma may present with similar clinical features [5,35,36,37].

Because many of these conditions share overlapping morphological characteristics, distinguishing malignant from benign lesions based solely on visual inspection can be challenging. This further emphasizes the importance of adjunctive diagnostic tools to support early and accurate diagnosis.

Clinically, oral mucosal melanoma presents as a pigmented, irregular, and asymmetric lesion larger than 6 mm, often exhibiting multiple color variations (black, brown, white, gray, purple, and red) and an uneven surface [2,8,16,19,25,38,39,40]. Oral lesions may occasionally be multifocal, with satellite lesions surrounding the primary tumor [8,40,41,42]. OMM may present with or without a radial growth phase [8,37]. Several case series have shown that up to one-third of oral melanomas are preceded by melanosis, which is thought to represent the radial growth phase before invasion into deeper tissues (vertical growth phase) occurs [8,17,20,23,41]. Approximately one-third of OMMs are amelanotic; these lesions are particularly challenging to diagnose, may lack a radial growth phase, and are frequently misdiagnosed as benign tumors or squamous cell carcinoma [8,15,29,43,44].

OMM is often initially asymptomatic but may become painful as the lesion enlarges [3,7,10,16,20]. As the disease progresses, additional symptoms such as ulceration, bleeding, paresthesia, and tooth mobility may develop [7,14,21,45]. Advanced cases may lead to regional lymph node involvement and distant metastases to the bones, lungs, and liver [2,4,6,10,14,21,40]. In a large series by Lian et al., the most common metastatic sites included regional lymph nodes (21.5%), lungs (21%), liver (18.5%), and distant lymph nodes (9%) [26].

OMM differs significantly from cutaneous melanoma in terms of epidemiology, genetic profile, clinical presentation, and response to therapy [5,12,29,46]. Recent studies employing “-omics” technologies have identified distinct genomic, molecular, and metabolic profiles in OMM, which may help explain differences in therapeutic response and clinical outcomes [12]. The current standard treatment for OMM remains surgical excision with clear margins [3,7,21,23].

Early lesions are frequently overlooked or misdiagnosed, as OMM may clinically resemble benign pigmented lesions, mucosal melanosis, or other forms of oral malignancy [23,39]. Although histopathology remains the gold standard for diagnosis, oral mucosal biopsies may be associated with practical limitations, including procedural complexity, reduced patient compliance, and potential postoperative complications [47,48,49]. In this context, non-invasive imaging techniques such as dermoscopy and reflectance confocal microscopy (RCM) represent valuable adjunctive tools that may facilitate the detection of early structural changes not visible during conventional clinical examination [43] (Table 1). These techniques may therefore contribute to earlier diagnosis, more accurate lesion assessment, and improved patient outcomes [24,43] (Table 1).

### 3.1. Dermoscopy

Dermoscopy, whether analog or digital, is a simple and effective tool for patient evaluation, using ×10 to ×100 magnification combined with a light source to visualize subsurface structures [50]. In the assessment of oral mucosal lesions, both contact and non-contact dermoscopy can be employed [51,52,53]. Polarized dermoscopy is often preferred, as it enables the visualization of deeper structures without the need for immersion fluids, which may be difficult to apply in the oral cavity [51,52,53]. For mucosal examination, dermatoscopes equipped with disposable caps or flexible contact plates are typically used to maintain hygiene and improve stability on moist surfaces [50]. However, dermoscopic examination of the oral cavity remains technically challenging due to limited access, patient movement, and the complex curvature of intraoral anatomical structures.

When used alongside clinical examination, dermoscopy represents an essential first-line diagnostic tool, particularly in dermato-oncology, where it enhances the early detection of both pigmented and non-pigmented lesions [8,50,54,55]. Nevertheless, its application in mucosal lesions is limited by the reduced availability of dermoscopic devices specifically designed for intraoral use [8].

Dermoscopic features of oral mucosal melanoma can be broadly classified into several categories. Pigment-related features include irregular diffuse pigmentation and the presence of multiple colors such as black, brown, blue, and gray [51,52,53]. Structural patterns include multicomponent patterns, structureless areas, and pseudo-networks [51,52,53]. Regression features may appear as a blue-whitish veil or gray areas, while vascular features include atypical or irregular vessels [51,52,53]. The coexistence of these findings increases the suspicion of malignancy.

Characteristic dermoscopic patterns suggestive of malignancy include irregular diffuse pigmentation, pseudo-network, regression structures, a blue-whitish veil, and atypical vascular patterns [11]. Matsushita et al. reported a case of labial melanoma displaying these features, including irregular diffuse pigmentation, a pseudo-network, regression structures, and a blue-whitish veil [56]. Olszewska et al. further identified this pattern as characteristic of melanoma not only in labial semi-mucosa but also in oral mucosal melanoma, aiding in differentiation from benign entities such as amalgam tattoos, which typically present as homogeneous, slightly grainy bluish areas [51].

Additional diagnostic clues include the presence of multiple colors (blue, black, gray, and brown), asymmetry of structures with irregular borders, abrupt interruption of the reticular pattern, and irregularly distributed heterogeneous dots and globules [7,11]. In a study of 140 pigmented lesions of the oral and anogenital mucosa, Blum et al. demonstrated that the presence of blue, gray, or white coloration in combination with a structureless pattern achieved a sensitivity of 100% and a specificity of 82.3% for melanoma [52]. Similarly, Lin et al. reported that 75% of melanomas of the oral and genital mucosa and semi-mucosa exhibited a multicomponent pattern, whereas only 25% presented a homogeneous pattern; however, this study was limited to an Asian population [53].

### 3.2. Reflectance Confocal Microscopy

Reflectance confocal microscopy (RCM) is a high-resolution, non-invasive imaging technique that enables real-time microscopic visualization of tissue structures. It facilitates bedside examination at a cellular level, supports biopsy guidance, when necessary, allows mapping of lesion extent, and aids in margin delineation [47,51,57,58,59,60,61]. The role of in vivo RCM in the diagnostic pathway of oral mucosal melanoma is supported by its ability to detect key cytoarchitectural alterations in oral mucosal neoplasia, thereby potentially reducing diagnostic delays [57,60]. In addition, it enables continuous, minimally invasive follow-up of patients over time [62]. RCM does not require the use of stains, contrast agents, or topical substances, as it relies on the intrinsic refractive properties of subcellular structures, minimizing the risk of allergic or irritant reactions [62]. Furthermore, as it does not involve ionizing radiation, it can be safely used in children and pregnant women [62].

RCM has been shown to improve diagnostic accuracy compared with conventional visual inspection [63]. Its primary clinical application is the evaluation of clinically or dermoscopically equivocal lesions with a low-to-moderate pre-test probability of malignancy [50]. This technique demonstrates a high degree of concordance with histopathology in a variety of skin conditions and has been widely implemented in dermatological practice [47,57,58,60,62]. RCM enables horizontal (en face) imaging of tissue, providing a visualization of skin layers up to approximately 200 µm in depth, and potentially deeper in mucosal tissues [64,65]. This horizontal imaging plane offers excellent correlation with clinical and dermoscopic findings, which is essential for accurate lesion assessment [64]. In addition, it allows evaluation of a larger field of view compared to traditional vertical histological sectioning [64].

Oral mucosa is particularly well suited for RCM due to its thin or absent cornified superficial layer, which permits deeper laser penetration and enhances resolution in the upper epithelial layers, allowing a detailed visualization of cellular morphology and stromal structures [57,66]. Similar to the skin, pigmentation in the oral mucosa is located at the dermoepithelial junction (DEJ), while keratinocytes appear with greater clarity, often showing bright, round nuclei in the superficial layers [57]. Studies evaluating both normal and pathological oral mucosa have demonstrated a strong correlation between RCM findings and conventional histology [57,60,62,67]. In normal mucosa, epithelial cells are uniform in size and shape, whereas malignant lesions exhibit architectural disorganization, cellular pleomorphism, and increased heterogeneity [57,68].

RCM reveals several characteristic features of oral mucosal melanoma that may support diagnosis. These findings can be grouped into key diagnostic criteria. Architectural disorganization is commonly observed, including loss of the normal epithelial–lamina propria junction and disruption of connective tissue papillae [48,53,57,64]. Cytological atypia is reflected by the presence of numerous bright atypical cells, including dendritic and round (pagetoid) cells within the epithelium [48,53,57,64]. A high density of basal hyper-reflective dendritic cells is frequently noted [48,53,57,64]. In addition, pagetoid spread of atypical melanocytes and sheet-like proliferation of atypical cells in the superficial lamina propria may be observed [48,53,57,64]. These features, particularly when present in combination, are highly suggestive of malignant melanocytic proliferation in the oral mucosa.

RCM provides detailed imaging of tissue characteristics, including cellular morphology, pigmentation, and vascular patterns, thereby supporting more accurate clinical decision-making [63]. Franceschini et al. proposed a diagnostic workflow for oral mucosal pigmented lesions, in which the loss of normal architecture at the epithelial–lamina propria junction and the presence of atypical cells are key features for distinguishing OMM from benign conditions [48]. Specifically, OMM is characterized by a high density of basal hyper-reflective dendritic cells, numerous bright pagetoid cells (typically round or fusiform with plump cytoplasm) within the epithelium, the disruption of connective tissue papillae, and the sheet-like proliferation of atypical cells within the superficial lamina propria [48,65,66].

Ardigò et al. reported that RCM findings in non-masticatory oral mucosa of the cheek showed a strong concordance with clinical diagnoses, with microscopic features closely correlating with histological observations [47]. These results support the feasibility of non-invasive, pre-histological evaluation of oral mucosal lesions using RCM and highlight its potential role in confirming or excluding malignant processes and guiding biopsy site selection.

RCM may help reduce the number of unnecessary biopsies in selected cases by improving diagnostic confidence and enabling more accurate targeting of biopsy sites [69]. A multicenter randomized trial demonstrated that the adjunctive use of RCM for suspicious lesions reduced unnecessary excisions while ensuring the timely removal of aggressive melanomas during initial clinical decision-making in referral centers [54,70]. This approach may be particularly valuable in routine clinical practice, where clinicians can assess lesions more precisely and determine the need for immediate tissue sampling [69].

Despite the high resolution and diagnostic potential of RCM and other non-invasive imaging techniques, several limitations must be considered in clinical practice, and histopathologic examination remains the gold standard for the definitive diagnosis of oral mucosal melanoma, particularly in primary lesions and suspected recurrences. Biopsy is especially required for amelanotic lesions, rapidly evolving lesions, or those displaying marked structural asymmetry or ulceration [49,63,71,72]. In these situations, imaging techniques should be regarded as supportive tools that assist clinical decision-making and help identify the most appropriate biopsy site, rather than as substitutes for histologic diagnosis.

Dermoscopy of oral mucosal lesions may also be technically challenging due to the anatomical configuration of the oral cavity, the limited availability of dedicated mucosal probes, and difficulties in achieving stable contact with moist surfaces. RCM, although capable of providing near-histologic resolution, requires specialized equipment, trained operators, and expertise in image interpretation [49,63,71,72]. Both image acquisition and interpretation require specific training and experience, and diagnostic accuracy may vary depending on the clinician’s level of expertise [49,63,71,72]. In addition, the limited penetration depth of RCM restricts the evaluation of deeper tumor components [49,63,71,72]. Furthermore, access to confocal devices remains limited in many clinical settings, which may hinder the widespread adoption of this technology in routine oral examinations.

Consequently, these techniques should be regarded as complementary tools that support, but do not replace, histopathological examination.

Several studies have evaluated the diagnostic performance of RCM in melanocytic lesions, demonstrating a high degree of concordance with histopathology. Reported sensitivity for melanoma diagnosis using RCM typically ranges from approximately 85% to 97%, while specificity varies between 70% and 88%, depending on the study population, diagnostic thresholds, and lesion characteristics [59,73]. The adjunctive use of RCM has been shown to significantly improve diagnostic accuracy compared with clinical and dermoscopic examination alone, while also reducing the number of unnecessary biopsies [59,73]. Although most available data are derived from cutaneous lesions, similar diagnostic principles are increasingly being applied to mucosal sites, supporting the role of RCM as a valuable complementary tool in the evaluation of oral mucosal melanoma.

The ability of RCM to reduce unnecessary excisions while ensuring the timely detection and management of aggressive melanomas highlights its significant value in clinical practice. This advantage is exemplified in the following case, in which RCM played a pivotal role in the diagnosis and management of recurrent OMM.

**Figure 1:**
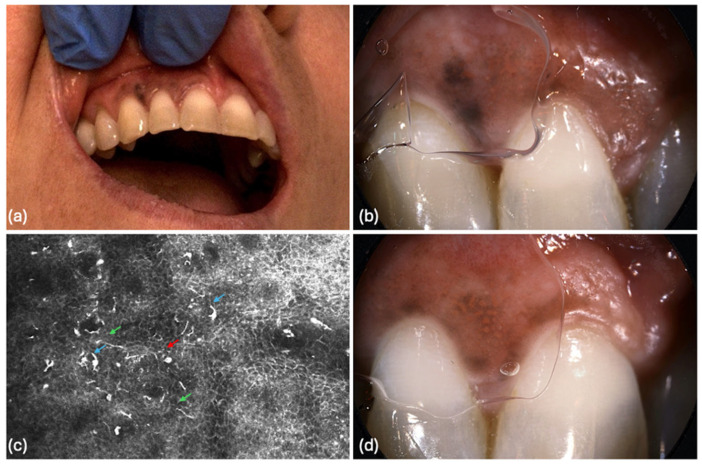
Oral mucosal melanoma (OMM) lesion. (**a**) Clinical image showing gingival mucosal hyperpigmentation presenting as an irregular macular lesion with variegated pigmentation intensity, more pronounced between the upper right lateral incisor and canine; (**b**) Dermoscopic image (×10) of the lesion located between the upper right lateral incisor and canine, revealing black hyperpigmented areas with architectural disarray, gray-whitish regression structures, and a surrounding brown pseudo-network; (**c**) Reflectance confocal microscopy (RCM) image (1.00 × 0.75 mm) at the dermoepidermal junction (DEJ), acquired using VivaScope^®^ 3000 (Caliber Imaging & Diagnostics, Rochester, NY, USA), demonstrating pagetoid cells characterized by round cells with bright cytoplasm and dark eccentric nuclei (red arrow), along with dendritic cells exhibiting short branches (blue arrows) and long, thin dendritic extensions (green arrows), as well as loss of connective tissue papillae architecture; (**d**) Dermoscopic image (×15) showing the lesion between the upper right central and lateral incisors, displaying a similar pseudo-network pattern with focal areas of darker brown pigment consolidation.

A 49-year-old female (Fitzpatrick skin type III) with a history of melanoma in situ of the hard palate presented to our department with asymptomatic black macules on the oral mucosa, without associated symptoms. Intraoral examination revealed an asymmetric pigmented lesion located on the right maxillary buccal vestibule (Figure 1).

RCM evaluation of the lesion demonstrated the presence of basal hyper-reflective dendritic cells, numerous pagetoid cells, loss of connective tissue papillae architecture, and atypical cellular features. These findings raised suspicion for multifocal recurrence and possible field cancerization, leading to a diagnosis of recurrent OMM. The diagnosis was subsequently confirmed by histopathological examination (Figure 1).

**Figure 2:**
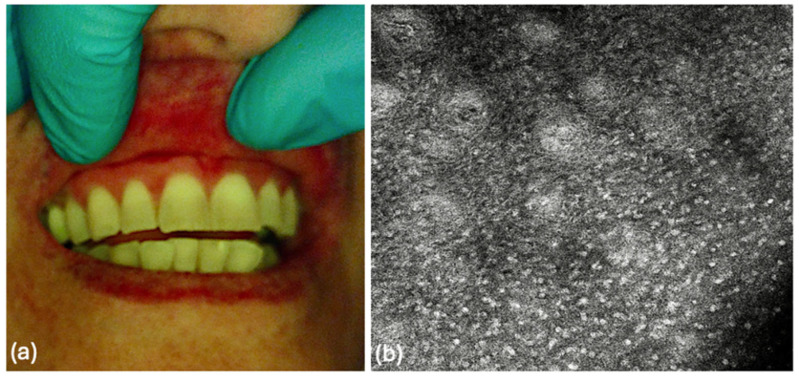
Follow-up after treatment with topical imiquimod 5%. (**a**) Clinical image obtained after three months of therapy, showing marked regression of gingival mucosal hyperpigmentation, with resolution of the previously observed irregular macular lesion between the upper right lateral incisor and canine and restoration of a more uniform gingival appearance; (**b**) Reflectance confocal microscopy (RCM) image (1.00 × 1.00 mm) at the spinous layer, acquired using VivaScope^®^ 3000, demonstrating a regular and homogeneous cellular pattern, with no evidence of pagetoid cells, atypical dendritic cells, or architectural disorganization of connective tissue papillae.

Based on these findings, topical imiquimod 5% was initiated (once daily, 5 days per week for six months), resulting in significant clinical improvement at three months (Figure 2). The therapeutic strategy was established within a multidisciplinary setting. Long-term surveillance with regular oral examinations over a five-year period has been planned.

In this case, RCM contributed to improved patient compliance, as the patient was more willing to adhere to follow-up when informed about the availability of non-invasive diagnostic methods [24,69]. Furthermore, RCM enabled continuous monitoring of the lesion over time through serial image acquisition, allowing the dynamic assessment of morphological changes [57,74]. This approach provided valuable insights into lesion progression or regression, which is particularly important in patients requiring long-term surveillance, such as those with a history of oral or cutaneous melanoma [57,74].

Despite its numerous advantages, reflectance confocal microscopy (RCM) also has several limitations, including restricted availability, operator dependency, and difficulties in accessing certain anatomical sites. These factors may limit its use in routine clinical practice. To improve usability, handheld confocal microscopes have been equipped with newly designed probes for intraoral imaging, tailored to the specific anatomical and topographical characteristics of the oral mucosa [48]. RCM using handheld devices is generally well tolerated by patients and allows high-quality image acquisition [58,62]. However, the current design and acquisition constraints limit evaluation primarily to the anterior two-thirds of the oral mucosa and do not allow adequate visualization of the hard palate [48].

**Figure 3:**
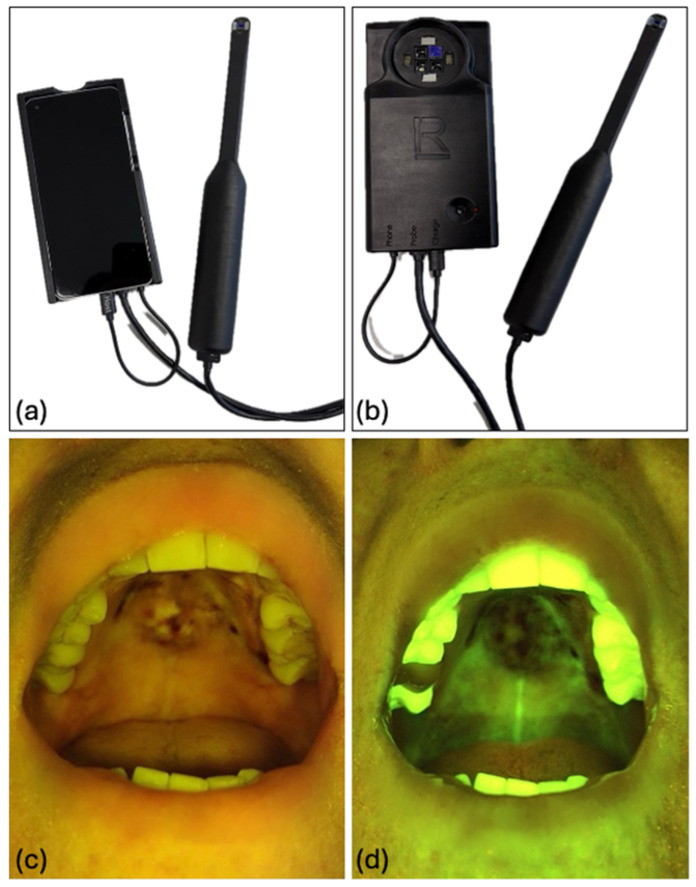
Multimodal intraoral imaging system and clinical appearance of OMM. (**a**,**b**) Prototype oral probe integrated with a multimodal intraoral imaging system designed for oral lesion assessment; (**c**,**d**) Clinical images of the oral mucosal melanoma located on the hard palate: (**c**) conventional clinical image without filters, showing a hyperpigmented lesion with interspersed whitish areas; (**d**) autofluorescence image demonstrating loss of autofluorescence signal within the lesion, corresponding to the areas of pigmentation.

The limited accessibility of the hard palate underscores the need for alternative approaches in the assessment of challenging anatomical areas. In our clinical practice, this limitation became evident in the following case, in which standard RCM was insufficient for comprehensive evaluation. A 50-year-old female patient (Fitzpatrick skin type II) with a history of chronic smoking presented with a progressively enlarging, asymptomatic nodular mass in the left laterocervical region. Clinical examination revealed a firm, non-mobile mass posterior to the left mastoid process. Intraoral assessment identified a diffuse, non-hemorrhagic pigmented lesion on the hard palate extending to the upper alveolar ridge (Figure 3).

Given the limitations of conventional RCM in this anatomical region, due to the lack of a flexible probe, a prototype multimodal intraoral imaging system with an oral probe designed for oral cancer detection was used as an alternative diagnostic approach (Figure 3, Video S1). Histopathological examination confirmed the diagnosis of melanoma with intraepithelial lentiginous melanocytic proliferation and regression areas, identifying the hard palate lesion as the primary site. Positron emission tomography–computed tomography (PET–CT) revealed no distant metastases other than the known laterocervical lesion, consistent with stage III oral melanoma.

### 3.3. Emerging Non-Invasive Optical Imaging Techniques

In addition to dermoscopy and reflectance confocal microscopy, several other non-invasive optical technologies are increasingly being investigated for the evaluation of oral mucosal lesions.

Autofluorescence-based imaging systems are light-based techniques used as non-invasive screening tools for oral potentially malignant disorders (OPMDs), by exploiting tissue-specific absorption and reflectance properties [74]. Cancerous and precancerous tissues exhibit altered fluorescence patterns due to metabolic and structural changes, which distinguish them from healthy tissues [74]. Intraoral probes utilizing autofluorescence technology enhance lesion visualization by detecting these abnormalities under specific excitation wavelengths [75].

Although this method demonstrates good sensitivity as a screening tool, its overall impact on diagnostic accuracy remains uncertain, as highlighted by a systematic review and meta-analysis by Kim et al. [74].

Optical coherence tomography (OCT) provides cross-sectional, high-resolution imaging of tissue microarchitecture and has demonstrated potential for detecting early epithelial and subepithelial alterations in oral malignancies [76,77,78,79,80].

Spectroscopy-based approaches, including Raman spectroscopy and diffuse reflectance spectroscopy, have shown promise in differentiating benign from malignant oral lesions through the analysis of tissue biochemical composition [81,82,83,84]. Furthermore, emerging portable and multimodal intraoral imaging platforms, integrating fluorescence imaging, high-resolution microendoscopy, and digital imaging technologies, may help overcome anatomical access limitations and facilitate real-time screening in clinical settings [81,82,83,84].

Although many of these techniques remain under investigation, they represent promising complementary tools that may further enhance the non-invasive diagnostic evaluation of oral mucosal lesions (Table 2).

Imaging techniques play a pivotal role in the diagnostic process by facilitating lesion detection, characterization, and more accurate clinical evaluation, ultimately supporting treatment planning. By improving diagnostic confidence, non-invasive technologies may reduce the risk of false-positive and false-negative findings, which is particularly important for the early detection of malignant lesions [63]. Nevertheless, limited visualization of certain anatomical sites, due to current device constraints, highlights the need for the development of more advanced and flexible probes to enable comprehensive intraoral assessment.

## 4. Conclusions

In conclusion, OMM is a rare but highly aggressive malignancy with a poor prognosis, largely attributable to delayed diagnosis and challenges in early detection. Increasing awareness among clinicians and improving familiarity with its clinical presentations and common anatomical sites are essential steps toward reducing diagnostic delay.

Although non-invasive imaging techniques have certain limitations, they represent valuable adjunctive tools in the differential diagnosis of pigmented lesions of the oral mucosa. These methods may enhance early detection, support longitudinal monitoring, and assist in identifying the most appropriate biopsy sites, thereby contributing to more informed clinical decision-making.

Nevertheless, histopathological examination remains indispensable for definitive diagnosis. The early recognition of OMM, supported by the appropriate use of non-invasive imaging techniques, is critical for optimizing patient management and improving survival outcomes. Further research is warranted to refine diagnostic criteria and to better define the role of these technologies in clinical practice.

## Figures and Tables

**Table 1:** Key characteristics of main non-invasive diagnostic techniques.

Technique	Resolution	Penetration Depth	Clinical Application	Advantages	Limitations
Dermoscopy	Low to moderate	Up to 1 mm	Examination of pigmented skin lesions. Differentiating melanomas from benign lesions.	Non-invasive and easy to use. Enhances diagnostic accuracy for pigmented lesions.	Limited to surface features. Cannot assess deeper structures or cellular details.
RCM	High	Up to 200 µm	Diagnosis of skin cancers (melanoma) and other pigmented lesions. Evaluation of skin, mucosal, and oral lesions.	Provides cellular-level resolution. Real-time, non-invasive imaging.	Limited penetration depth. Requires trained operators and specialized equipment.

**Table 2:** Summary of representative studies on non-invasive imaging techniques in oral mucosal melanoma and related pigmented lesions.

Author (Year)	Study Type	Modality	Focus	Main Findings	Clinical Relevance
Franceschini et al. (2023) [48]	Review	RCM	Oral lesions	RCM identifies cytoarchitectural changes and supports diagnosis of oral lesions	Diagnostic support
Liu et al. (2021) [60]	Review	RCM	OPMDs	RCM correlates well with histopathology and allows non-invasive evaluation	Early detection
Maher et al. (2017) [61]	Clinical study	RCM	Lip melanoma	High concordance between RCM and histopathology	Differential diagnosis
Blum et al. (2011) [52]	Multicenter study	Dermoscopy	Mucosal lesions	Specific dermoscopic patterns associated with melanoma	Screening/differentiation
Kim et al. (2020) [74]	Meta-analysis	Autofluorescence	OPMDs	High sensitivity but limited specificity	Screening tool
Gentile et al. (2017) [77]	Systematic review	OCT	Oral lesions	OCT provides high-resolution cross-sectional imaging	Structural assessment
Hanna et al. (2024) [81]	Review	Raman spectroscopy	Oral cancer	Spectroscopy detects biochemical changes in malignant tissue	Early diagnosis
Kolpakov et al. (2023) [83]	Clinical study	Diffuse reflectance spectroscopy	Oral mucosa	Effective in detecting precancerous lesions in vivo	Non-invasive screening
Singh et al. (2016) [84]	Review	Multimodal imaging	Oral cancer	Combined optical methods improve diagnostic accuracy	Real-time assessment

## Data Availability

No new data were created or analyzed in this study. Data sharing is not applicable to this article.

## Supplementary Materials

The following supporting information can be downloaded at: https://www.mdpi.com/article/10.3390/diagnostics16071030/s1. Video S1. Evaluation of oral mucosal lesions using the prototype oral probe integrated into a multimodal intraoral imaging system.

## Abbreviations

The following abbreviations are used in this manuscript:

OMM	Oral mucosal melanoma
RCM	Reflectance confocal microscopy
CM	Cutaneous melanoma
MAPK	Mitogen-activated protein kinase
DEJ	Dermoepidermal junction
PET-CT	Positron emission tomography–computed tomography
OPMDs	Oral potentially malignant disorders

## References

[1] Zito P.M., Brizuela M., Mazzoni T. (2023). Oral Melanoma. StatPearls.

[2] Misra S.R., Tripathy U.R., Das R., Mohanty N. (2021). Oral malignant melanoma: A rarity!. BMJ Case Rep..

[3] Jasper P., Jungbauer W.N., Poupore N.S., Nguyen S.A., Howell J., Neville B.W., Day T.A. (2022). Mucosal Melanoma In Situ of the Oral Cavity: A Case Report and Systematic Review of the Literature. Turk. Arch. Otolaryngol..

[4] Thomas P.S., Babu G.S., Anusha R.L., Shetty S. (2012). Oral malignant melanoma—An unusual presentation. Gerodontology.

[5] López F., Rodrigo J.P., Cardesa A., Triantafyllou A., Devaney K.O., Mendenhall W.M., Haigentz M., Strojan P., Pellitteri P.K., Bradford C.R. (2016). Update on primary head and neck mucosal melanoma. Head Neck.

[6] Uchiyama Y., Sasai T., Nakatani A., Shimamoto H., Tsujimoto T., Kreiborg S., Murakami S. (2021). Distant metastasis from oral cavity-correlation between histopathology results and primary site. Oral Radiol..

[7] Ashok S., Damera S., Ganesh S., Karri R. (2020). Oral malignant melanoma. J. Oral Maxillofac. Pathol..

[8] Warszawik-Hendzel O., Słowińska M., Olszewska M., Rudnicka L. (2014). Melanoma of the oral cavity: Pathogenesis, dermoscopy, clinical features, staging and management. J. Dermatol. Case Rep..

[9] Lambertini M., Patrizi A., Fanti P., Melotti B., Caliceti U., Magnoni C., Misciali C., Baraldi C., Ravaioli G., Dika E. (2018). Oral melanoma and other pigmentations: When to biopsy?. J. Eur. Acad. Dermatol. Venereol..

[10] Singh D., Pandey P., Singh M., Kudva S. (2019). Prevalence of malignant melanoma in anatomical sites of the oral cavity: A meta-analysis. J. Oral Maxillofac. Pathol..

[11] Dika E., Lambertini M., Pellegrini C., Veronesi G., Melotti B., Riefolo M., Sperandi F., Patrizi A., Ricci C., Mussi M. (2021). Cutaneous and Mucosal Melanomas of Uncommon Sites: Where Do We Stand Now?. J. Clin. Med..

[12] Santeufemia D.A., Palmieri G., Miolo G., Colombino M., Doro M.G., Frogheri L., Paliogiannis P., Capobianco G., Madonia M., Cossu A. (2023). Current Trends in Mucosal Melanomas: An Overview. Cancers.

[13] Boulaadas M., Benazzou S., Mourtada F., Sefiani S., Nazih N., Essakalli L., Kzadri M. (2007). Primary oral malignant melanoma. J. Craniofacial Surg..

[14] Meleti M., Leemans C.R., Mooi W.J., Vescovi P., van der Waal I. (2007). Oral malignant melanoma: A review of the literature. Oral Oncol..

[15] Soares C.D., Carlos R., de Andrade B.A.B., Cunha J.L.S., Agostini M., Romañach M.J., Hernandez-Guerrero J.C., Mosqueda-Taylor A., de Almeida O.P., Jorge J. (2021). Oral Amelanotic Melanomas: Clinicopathologic Features of 8 Cases and Review of the Literature. Int. J. Surg. Pathol..

[16] Alawi F. (2013). Pigmented lesions of the oral cavity: An update. Dent. Clin. N. Am..

[17] Topić B., Mašić T., Radović S., Lincender I., Muhić E. (2017). Primary Oral Mucosal Melanomas—Two Case Reports and Comprehensive Literature Review. Acta Clin. Croat..

[18] Rambhia P.H., Stojanov I.J., Arbesman J. (2019). Predominance of oral mucosal melanoma in areas of high mechanical stress. J. Am. Acad. Dermatol..

[19] Hajar-Serviansky T., Gutierrez-Mendoza D., Galvan I.L., Lammoglia-Ordiales L., Mosqueda-Taylor A., de Lourdes Hernandez-Cázares M., Toussaint-Caire S. (2012). A case of oral mucosal melanoma. Clinical and dermoscopic correlation. J. Dermatol. Case Rep..

[20] Aguas S.-C., Quarracino M.-C., Lence A.-N., Lanfranchi-Tizeira H.-E. (2009). Primary melanoma of the oral cavity: Ten cases and review of 177 cases from literature. Med. Oral Patol. Oral Cir. Bucal.

[21] Mohan M., Sukhadia V.Y., Pai D., Bhat S. (2013). Oral malignant melanoma: Systematic review of literature and report of two cases. Oral Surg. Oral Med. Oral Pathol. Oral Radiol..

[22] Hatch C.L. (2005). Pigmented lesions of the oral cavity. Dent. Clin. N. Am..

[23] Femiano F., Lanza A., Buonaiuto C., Gombos F., Di Spirito F., Cirillo N. (2008). Oral malignant melanoma: A review of the literature. J. Oral Pathol. Med..

[24] Wushou A., Zhao Y.-J. (2015). The management and site-specific prognostic factors of primary oral mucosal malignant melanoma. J. Craniofac. Surg..

[25] Barata B., Freitas F., Vilares M., Caramês J. (2024). Oral mucosal melanoma: A systematic review of case reports and case series. J. Oral Maxillofac. Surg. Med. Pathol..

[26] Lian B., Cui C.L., Zhou L., Song X., Zhang X.S., Wu D., Si L., Chi Z.H., Sheng X.N., Mao L.L. (2017). The natural history and patterns of metastases from mucosal melanoma: An analysis of 706 prospectively-followed patients. Ann. Oncol..

[27] Lamichhane N.S., An J., Liu Q., Zhang W. (2015). Primary malignant mucosal melanoma of the upper lip: A case report and review of the literature. BMC Res. Notes.

[28] Tyrrell H., Payne M. (2018). Combatting mucosal melanoma: Recent advances and future perspectives. Melanoma Manag..

[29] Abati S., Sandri G.F., Finotello L., Polizzi E. (2024). Differential Diagnosis of Pigmented Lesions in the Oral Mucosa: A Clinical Based Overview and Narrative Review. Cancers.

[30] Song H., Wang L., Lyu J., Wu Y., Guo W., Ren G. (2017). Loss of nuclear BAP1 expression is associated with poor prognosis in oral mucosal melanoma. Oncotarget.

[31] Cui C., Lian B., Zhou L., Song X., Zhang X., Wu D., Chi Z., Si L., Sheng X., Kong Y. (2018). Multifactorial Analysis of Prognostic Factors and Survival Rates Among 706 Mucosal Melanoma Patients. Ann. Surg. Oncol..

[32] Gu G.M., Epstein J.B., Morton T.H. (2003). Intraoral melanoma: Long-term follow-up and implication for dental clinicians. A case report and literature review. Oral Surg. Oral Med. Oral Pathol. Oral Radiol. Endodontol..

[33] Hicks M., Flaitz C. (2000). Oral mucosal melanoma: Epidemiology and pathobiology. Oral Oncol..

[34] Rapidis A.D., Apostolidis C., Vilos G., Valsamis S. (2003). Primary malignant melanoma of the oral mucosa. J. Oral Maxillofac. Surg..

[35] Kauzman A., Pavone M., Blanas N., Bradley G. (2004). Pigmented lesions of the oral cavity: Review, differential diagnosis, and case presentations. J. Can. Dent. Assoc..

[36] Eisen D. (2000). Disorders of pigmentation in the oral cavity. Clin. Dermatol..

[37] Gondak R., da Silva-Jorge R., Jorge J., Lopes M., Vargas P. (2012). Oral pigmented lesions: Clinicopathologic features and review of the literature. Med. Oral Patol. Oral Y Cir. Bucal.

[38] Sen S., Sen S., Kumari M.G., Khan S., Singh S. (2021). Oral Malignant Melanoma: A Case Report. Prague Med. Rep..

[39] Feller L., Khammissa R.A.G., Lemmer J. (2017). A Review of the Aetiopathogenesis and Clinical and Histopathological Features of Oral Mucosal Melanoma. Sci. World J..

[40] Meleti M., Vescovi P., Mooi W.J., van der Waal I. (2008). Pigmented lesions of the oral mucosa and perioral tissues: A flow-chart for the diagnosis and some recommendations for the management. Oral Surg. Oral Med. Oral Pathol. Oral Radiol. Endodontol..

[41] Tacastacas J.D., Bray J., Cohen Y.K., Arbesman J., Kim J., Koon H.B., Honda K., Cooper K.D., Gerstenblith M.R. (2014). Update on primary mucosal melanoma. J. Am. Acad. Dermatol..

[42] Patel P.B., Wright J.M., Kang D.R., Cheng Y.-S.L. (2018). Longitudinal clinicopathologic data of the progression of oral mucosal melanoma-report of 2 cases and literature review. Oral Surg. Oral Med. Oral Pathol. Oral Radiol..

[43] Kaul S., Kumar P. (2013). Significance of early detection of oral malignant melanoma in improving prognosis. S. Asian J. Cancer.

[44] Bansal S.P., Dhanawade S.S., Arvandekar A.S., Mehta V., Desai R.S. (2022). Oral Amelanotic Melanoma: A Systematic Review of Case Reports and Case Series. Head Neck Pathol..

[45] de Castro M.S., de Assis Reis B.S., Nogueira D.A., de Carli M.L., Costa Hanemann J.A., Costa Pereira A.A., de Almeida O.P., Sperandio F.F. (2017). Primary oral melanoma: A clinicopathologic review and case presentation. Quintessence Int..

[46] Chatzistefanou I., Kolokythas A., Vahtsevanos K., Antoniades K. (2016). Primary mucosal melanoma of the oral cavity: Current therapy and future directions. Oral Surg. Oral Med. Oral Pathol. Oral Radiol..

[47] Ardigo M., Donadio C., Franceschini C., Catricalà C., Agozzino M. (2015). Interest of reflectance confocal microscopy for inflammatory oral mucosal diseases. J. Eur. Acad. Dermatol. Venereol..

[48] Franceschini C., Mandel V.D., Peterson G., Manciocco V., Guitera P., Rajadhyaksha M., Ardigò M. (2023). Role of reflectance confocal microscopy for in vivo investigation of oral disorders: White, red and pigmented lesions. Exp. Dermatol..

[49] Lingen M.W., Abt E., Agrawal N., Chaturvedi A.K., Cohen E., D’sOuza G., Gurenlian J., Kalmar J.R., Kerr A.R., Lambert P.M. (2017). Evidence-based clinical practice guideline for the evaluation of potentially malignant disorders in the oral cavity: A report of the American Dental Association. J. Am. Dent. Assoc..

[50] Mandel V.D., Ardigò M. (2023). Non-Invasive Diagnostic Techniques in Dermatology. J. Clin. Med..

[51] Olszewska M., Banka A., Gorska R., Warszawik O. (2008). Dermoscopy of pigmented oral lesions. J. Dermatol. Case Rep..

[52] Blum A., Simionescu O., Argenziano G., Braun R., Cabo H., Eichhorn A., Kittler H. (2011). Dermoscopy of pigmented lesions of the mucosa and the mucocutaneous junction: Results of a multicenter study by the International Dermoscopy Society (IDS). Arch. Dermatol..

[53] Lin J., Koga H., Takata M., Saida T. (2009). Dermoscopy of pigmented lesions on mucocutaneous junction and mucous membrane. Br. J. Dermatol..

[54] Soglia S., Pérez-Anker J., Guede N.L., Giavedoni P., Puig S., Malvehy J. (2022). Diagnostics Using Non-Invasive Technologies in Dermatological Oncology. Cancers.

[55] Orzan O.A., Dorobanțu A.M., Gurău C.D., Ali S., Mihai M.M., Popa L.G., Giurcăneanu C., Tudose I., Bălăceanu B. (2023). Challenging Patterns of Atypical Dermatofibromas and Promising Diagnostic Tools for Differential Diagnosis of Malignant Lesions. Diagnostics.

[56] Matsushita S., Kageshita T., Ishihara T. (2005). Comparison of dermoscopic and histopathological findings in a mucous melanoma of the lip. Br. J. Dermatol..

[57] Franceschini C., Persechino F., Ardigò M. (2020). In Vivo Reflectance Confocal Microscopy in General Dermatology: How to Choose the Right Indication. Dermatol. Pract. Concept..

[58] Ardigo M., Longo C., Gonzalez S. (2016). The International Confocal Working Group Inflammatory Skin Diseases Project. Multicentre study on inflammatory skin diseases from The International Confocal Working Group: Specific confocal microscopy features and an algorithmic method of diagnosis. Br. J. Dermatol..

[59] Dinnes J., Deeks J.J., Saleh D., Chuchu N., E Bayliss S., Patel L., Davenport C., Takwoingi Y., Godfrey K., Matin R.N. (2018). Reflectance confocal microscopy for diagnosing cutaneous melanoma in adults. Cochrane Database Syst. Rev..

[60] Liu W., Yang X., Shen Z., Shi L. (2021). The characteristics and prospects of reflectance confocal microscopy for noninvasive diagnosis of oral potentially malignant disorders. Oral Oncol..

[61] Maher N.G., Solinas A., Scolyer R.A., Guitera P. (2017). In vivo reflectance confocal microscopy for evaluating melanoma of the lip and its differential diagnoses. Oral Surg. Oral Med. Oral Pathol. Oral Radiol..

[62] Contaldo M., Agozzino M., Moscarella E., Esposito S., Serpico R., Ardigò M. (2013). In vivo characterization of healthy oral mucosa by reflectance confocal microscopy: A translational research for optical biopsy. Ultrastruct. Pathol..

[63] Rajadhyaksha M., Marghoob A., Rossi A., Halpern A.C., Nehal K.S. (2017). Reflectance confocal microscopy of skin in vivo: From bench to bedside. Lasers Surg. Med..

[64] Longo C., Zalaudek I., Argenziano G., Pellacani G. (2012). New directions in dermatopathology: In vivo confocal microscopy in clinical practice. Dermatol. Clin..

[65] Uribe P., Collgros H., Scolyer R.A., Menzies S.W., Guitera P. (2017). In Vivo Reflectance Confocal Microscopy for the Diagnosis of Melanoma and Melanotic Macules of the Lip. JAMA Dermatol..

[66] Cinotti E., Labeille B., Cambazard F., Perrot J.-L. (2016). Confocal Microscopy for Special Sites and Special Uses. Dermatol. Clin..

[67] García-Hernández A., Roldán-Marín R., Iglesias-Garcia P., Malvehy J. (2013). In Vivo Noninvasive Imaging of Healthy Lower Lip Mucosa: A Correlation Study between High-Definition Optical Coherence Tomography, Reflectance Confocal Microscopy, and Histology. Dermatol. Res. Pract..

[68] Agozzino M., Bhasne P., Franceschini C., Vincenza G., Catricalà C., Ardigò M. (2014). Noninvasive, in vivo assessment of oral squamous cell carcinoma. Br. J. Dermatol..

[69] Arya S., Chaukar D., Pai P. (2012). Imaging in oral cancers. Indian J. Radiol. Imaging.

[70] Pellacani G., Farnetani F., Ciardo S., Chester J., Kaleci S., Mazzoni L., Longo C. (2022). Effect of Reflectance Confocal Microscopy for Suspect Lesions on Diagnostic Accuracy in Melanoma: A Randomized Clinical Trial. JAMA Dermatol..

[71] Pellacani G., Longo C., Malvehy J., Puig S., Carrera C., Segura S., Bassoli S., Seidenari S. (2008). In vivo confocal microscopic and histopathologic correlations of dermoscopic features in 202 melanocytic lesions. Arch. Dermatol..

[72] Guitera P., Pellacani G., Crotty K.A., Scolyer R.A., Li L.-X.L., Bassoli S., Vinceti M., Rabinovitz H., Longo C., Menzies S.W. (2010). The impact of in vivo reflectance confocal microscopy on the diagnostic accuracy of lentigo maligna and equivocal pigmented and nonpigmented macules of the face. J. Investig. Dermatol..

[73] Pellacani G., Guitera P., Longo C., Avramidis M., Seidenari S., Menzies S. (2007). The impact of in vivo reflectance confocal microscopy for the diagnostic accuracy of melanoma and equivocal melanocytic lesions. J. Investig. Dermatol..

[74] Kim D.H., Kim S.W., Hwang S.H. (2020). Autofluorescence imaging to identify oral malignant or premalignant lesions: Systematic review and meta-analysis. Head Neck.

[75] Lima I.F.P., Brand L.M., de Figueiredo J.A.P., Steier L., Lamers M.L. (2021). Use of autofluorescence and fluorescent probes as a potential diagnostic tool for oral cancer: A systematic review. Photodiagn. Photodyn. Ther..

[76] Wilder-Smith P., Jung W., Brenner M., Osann K., Beydoun H., Messadi D., Chen Z. (2004). In vivo optical coherence tomography for the diagnosis of oral malignancy. Lasers Surg. Med..

[77] Gentile E., Maio C., Romano A., Laino L., Lucchese A. (2017). The potential role of in vivo optical coherence tomography for evaluating oral soft tissue: A systematic review. J. Oral Pathol. Med..

[78] Kim D.H., Kim S.W., Hwang S.H. (2023). Efficacy of optical coherence tomography in the diagnosing of oral cancerous lesion: Systematic review and meta-analysis. Head Neck.

[79] Katkar R.A., Tadinada S.A., Amaechi B.T., Fried D. (2018). Optical Coherence Tomography. Dent. Clin. N. Am..

[80] Chen X., Zhou G., Zhang J. (2024). Optical coherence tomography: Promising imaging technique for the diagnosis of oral mucosal diseases. Oral Dis..

[81] Hanna K., Asiedu A.-L., Theurer T., Muirhead D., Speirs V., Oweis Y., Abu-Eid R. (2024). Advances in Raman spectroscopy for characterising oral cancer and oral potentially malignant disorders. Expert Rev. Mol. Med..

[82] Maryam S., Nogueira M.S., Gautam R., Krishnamoorthy S., Sekar S.K.V., Kho K.W., Lu H., Ni Riordain R., Feeley L., Sheahan P. (2022). Label-Free Optical Spectroscopy for Early Detection of Oral Cancer. Diagnostics.

[83] Kolpakov A.V., Moshkova A.A., Melikhova E.V., Sokolova D.Y., Muravskaya N.P., Samorodov A.V., Kopaneva N.O., Lukina G.I., Abramova M.Y., Mamatsashvili V.G. (2023). Diffuse Reflectance Spectroscopy of the Oral Mucosa: In Vivo Experimental Validation of the Precancerous Lesions Early Detection Possibility. Diagnostics.

[84] Singh S., Ibrahim O., Byrne H.J., Mikkonen J.W., Koistinen A.P., Kullaa A.M., Lyng F.M. (2016). Recent advances in optical diagnosis of oral cancers: Review and future perspectives. Head Neck.

